# Determination of the Halogenated Skeleton Constituents of the Marine Demosponge *Ianthella basta*

**DOI:** 10.3390/md15020034

**Published:** 2017-02-10

**Authors:** Susanne Ueberlein, Susanne Machill, Peter J. Schupp, Eike Brunner

**Affiliations:** 1 Bioanalytical Chemistry, Fachrichtung Chemie und Lebensmittelchemie, TU Dresden, 01062 Dresden, Germany; susanne.ueberlein@chemie.tu-dresden.de (S.U.); susanne.machill@chemie.tu-dresden.de (S.M.); 2 Environmental Biochemistry, Institute of Chemistry and Biology of the Marine Environment, Carl-von-Ossietzky University Oldenburg, 26382 Wilhelmshaven, Germany; peter.schupp@uni-oldenburg.de

**Keywords:** demosponges, *Ianthella basta*, skeletons, spongin, amino acid composition, halogenated amino acids, GC-MS

## Abstract

Demosponges of the order Verongida such as *Ianthella basta* exhibit skeletons containing spongin, a collagenous protein, and chitin. Moreover, Verongida sponges are well known to produce bioactive brominated tyrosine derivatives. We recently demonstrated that brominated compounds do not only occur in the cellular matrix but also in the skeletons of the marine sponges *Aplysina cavernicola* and *I. basta*. Further investigations revealed the amino acid composition of the skeletons of *A. cavernicola* including the presence of several halogenated amino acids. In the present work, we investigated the skeletal amino acid composition of the demosponge *I. basta*, which belongs to the Ianthellidae family, and compared it with that of *A. cavernicola* from the Aplysinidae family. Seventeen proteinogenic and five non-proteinogenic amino acids were detected in *I. basta*. Abundantly occurring amino acids like glycine and hydroxyproline show the similarity of *I. basta* and *A. cavernicola* and confirm the collagenous nature of their sponging fibers. We also detected nine halogenated tyrosines as an integral part of *I. basta* skeletons. Since both sponges contain a broad variety of halogenated amino acids, this seems to be characteristic for Verongida sponges. The observed differences of the amino acid composition confirm that spongin exhibits a certain degree of variability even among the members of the order Verongida.

## 1. Introduction

The oceans represent the biggest habitat on earth, which is however, not yet fully explored. Consequently, the marine habitat is one of increasing interest for the research and further exploration of this understudied environment. For example, a huge diversity of bioactive natural products is found in marine organisms. To date, approximately 28,000 of these marine natural products have been discovered, and sponges are the main producers of such compounds [1,2].

Sponges (Porifera), representing one of the oldest metazoans, are often a prominent component of benthic communities [3]. They can be found in polar, temperate and tropical regions [4,5]. Sponges are sessile filterfeeders which cannot escape or actively fight predators [6,7]. Therefore, they developed morphological and chemical defense mechanisms, providing them with protection against various predators [8,9,10,11,12,13].

Based on their skeletal composition, sponges can be divided into three classes: calcareous sponges (Calcarea), glass sponges (Hexactinellida), and demosponges (Demospongia) [14]. Recently, a fourth class, the Homoscleromorpha, was phylogenetically defined [15,16].

Demospongiae represent the largest class of sponges [17]. Their skeletons are made from proteins [18], polysaccharides [19,20], and/or inorganic components such as siliceous spicules [14,21]. Demosponges are subdivided into three orders: Dendroceratida, Dictyoceratida, and Verongida. The skeletons of all of these orders exhibit spongin, a collagenous protein [22].

Till now, spongin is not really well defined. Nevertheless, it has remarkable properties. Spongin is considered to be a halogenated scleroprotein [22] of collagenous character. Spongin fibers are more resistant than collagen fibers against enzymatic digestion [23]. Exposito et al. showed in genomic and cDNA studies that spongin possesses the typical collagen sequence motif Gly-Xaa-Yaa [24]. Glycine is thus the most abundant amino acid found in spongin. Apart from glycine, the amino acids serine, arginine, lysine, valine and cysteine are reported to occur in spongin [25]. Additionally, Ehrlich et al. showed that the polysaccharide chitin appears as an integral skeleton component in Verongida sponge skeletons [19,20,26], forming a chitin scaffold of the same overall morphology as the integer skeleton.

It is not yet clear how spongin is connected with these chitin scaffolds in sponge skeletons. However, there are a number of examples of so-called chitin-protein complexes in nature showing that proteins are often strongly—mostly covalently—bound to chitin [27,28,29]. Hackman suggested that proteins are covalently bound to chitin in different insects and crustaceans [27]. Furthermore, Blackwell and Weih developed a model for the three-dimensional structure of an insect chitin-protein complex with chitin fibrils surrounded by layers of proteins [28]. These examples lead to the assumption that spongin in the skeletons of the Verongida sponges is also strongly—maybe covalently—bound to the chitin scaffolds.

Furthermore, sponges of the order Verongida are well-known for the biosynthesis of characteristic bioactive natural products, especially brominated tyrosine derivatives like bastadins from *Ianthella basta* (*I. basta*) [30,31,32,33], aerothionin from *Aplysina cavernicola* (*A. cavernicola*) [34], or psammaplins from *Aplysinella* sp. [11]. These compounds are biosynthesized from brominated tyrosines [35]. Initial investigations show that brominated substances are present even in the sponge skeletons [36,37]. Since brominated tyrosines can inhibit chitinase activity [38], these compounds could have a protective effect preventing undesired skeletal degradation.

Until now the full amino acid composition of sponges was only determined for three species. This includes the commercial unbleached sponge *Hippospongia equina* (*H. equina*) and the bath sponge *Spongia officinalis obliqua* (*S. officinalis obliqua*). Both of the sponges belong to the order Dictyoceratida [39,40]. Furthermore, we have recently determined the skeletal amino acid composition of the Verongida sponge *A. cavernicola* [41]. These studies revealed the presence of halogenated tyrosines in addition to non-halogenated amino acids. 3,5-Diiodotyrosine was found in *H. equina* [39], while 3-Monoiodo-tyrosine, 3,5-Diiodotyrosine, and 3,5-Dibromotyrosine could be detected in *S. officinalis obliqua* [40]. Compared to these two Dictyoceratida sponges, the Verongida sponge *A. cavernicola* exhibits a surprising variety of halogenated, i.e., brominated, iodated, and chlorinated amino acids [41].

The goal of the present study is to examine the amino acid composition of the sponge skeleton of another Verongida sponge and to compare it with the other three known amino acid compositions of sponges. The results should reveal whether or not there is a significant difference between the Verongida sponges from two different families. This may encourage future investigations of further sponge species.

The order Verongida comprises four families distinguished mainly by the structure and composition of their spongin fibers [42,43]. Aplysinidae is the largest verongid family (63 species from three genera: *Aplysina*, *Verongula*, and *Aiolochroia*). This family is defined by an anastomosing fiber skeleton with both pith and bark elements [18]. Ianthellidae is the second largest verongid family (12 species from three genera: *Ianthella*, *Anomoianthella*, and *Hexadella*). This family is distinguished from other Verongida families by the presence of eurypylous choanocyte chambers. Aplysinellidae consists of nine species from three genera (*Aplysinella*, *Porphyria*, and *Suberea*). It is defined by a dendritic fiber skeleton with both pith and bark elements. Pseudoceratinidae consists of four species from a single genus (*Pseudoceratina*) and is defined by a dendritic fiber skeleton with only pith elements. We have chosen *I. basta* as a characteristic representative of the Ianthellidae family, which represents the second largest among the four Verongida sponge families.

Based on previous work on *A. cavernicola* [41], we optimized the methods for the effective isolation of the sponge skeletons of *I. basta* and the complete extraction of amino acids from the skeletons with Ba(OH)_2_. The determined amino acid composition of *I. basta* was finally compared with the amino acid composition of *A. cavernicola* skeletons as well as with the amino acid composition of the Dictyoceratida sponges *H. equina* and *S. officinalis obliqua*.

## 2. Results and Discussion

### 2.1. General Chemical Characterization of the I. basta Skeleton and Comparison with A. cavernicola

**Figure 1:**
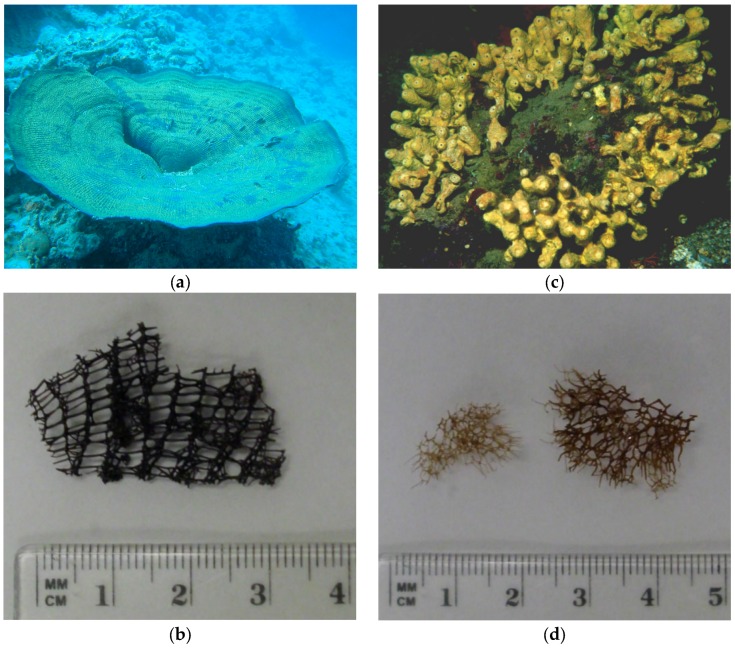
Underwater and light microscopic images of the sponges and their isolated skeletons after extraction; (**a**,**b**) *I. basta*; (**c**,**d**) *A. cavernicola* (photos: (**a**) Peter Schupp, (**c**) Carsten Thoms).

Morphological differences between the skeletons of the two Verongida sponges *I. basta* and *A. cavernicola* are shown in underwater and light microscopic images (Figure 1a–d). *I. basta* grows in large fan or funnel shapes (Figure 1a) which is reflected in its planar, netlike skeleton structure (Figure 1b). *A. cavernicola* grows thick and incrusting with numerous short tubes (Figure 1c), which is supported by its three-dimensional skeleton (Figure 1d).

**Figure 2:**
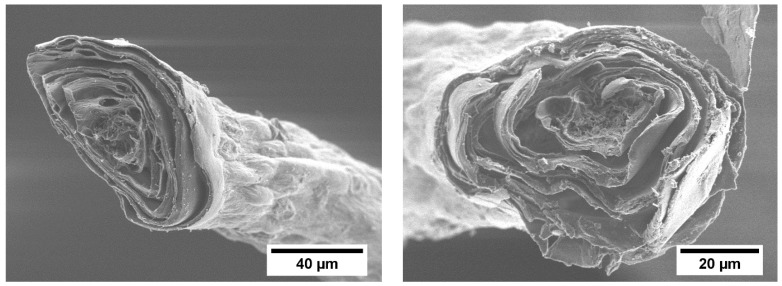
Scanning electron microscopy (SEM) images of the skeletons of *I. basta.*

The SEM images of the isolated *I. basta* skeletons (Figure 2) reveal a fiber system. These fibers consist of several concentric layers similar to the structural organization previously described for *A. cavernicola* skeletons (see [44]). The central channel of the fiber is filled with the pith, a porous material.

Table 1 summarizes various chemical parameters from the two sponges. Interestingly, the amount of skeletal material related to the entire sponge is more than one order of magnitude higher in *I. basta* than in *A. cavernicola*. An explanation for the higher amount of skeletal material in *I. basta* might be a need for mechanical stability because of its two-dimensional skeleton structure and the large size of the sponges (more than 2 m maximum diameter) [45].

The chitin content in *I. basta* skeletons is also higher than in *A. cavernicola*. Campbell [46] showed that chitin is responsible for the flexibility and not for the strength of chitin-protein complexes of insects. If this conclusion also applies here, it would lead to the assumption that the sponge skeleton of *I. basta* has to be more flexible than the skeletons of *A. cavernicola*. Given that the sponges can be found at depths as shallow as 5 m, where wave exposure certainly affects large sponges, a flexible skeleton might be a prerequisite to successfully colonize and persist on shallow reef slopes.

Furthermore, the percentage of saccharides in the skeletons other than chitin (estimated by the so-called resorcin method [47], see Materials and Methods) is also higher in *I. basta*, but in general much lower than the amount of chitin.

Ehrlich et al. [48] reported the presence of amorphous silicate and crystalline calcium carbonate in the form of aragonite as skeletal components of the Verongida sponge *Verongula giganteum*. In order to test this possibility for *I. basta*, the calcium and silicon contents of the skeletons were determined by ICP-OES. The calcium concentrations are low. Assuming that the calcium in the skeletons occurs as calcium carbonate, an amount of ca. 4% for *I. basta* and ca. 1% for *A. cavernicola* can be estimated. The silicon content is below the detection limit. These results show that both sponges, *I. basta* and *A. cavernicola*, do not exhibit the silica-aragonite-chitin-biocomposites found in *Verongula giganteum*.

The content of sulfur is of the same order for the skeletons of both samples. EDX measurements confirm that chlorine and iodine are present in both skeletons in addition to bromine. The bromine content (determined as described previously in [44]) is significantly higher in *I. basta* skeletons than in *A. cavernicola*.

Finally, we estimated the protein content of the skeletons as a remaining fraction after the consideration of the other analyses. It is significantly higher in *A. cavernicola* than in *I. basta*—mainly due to the higher amount of chitin in *I. basta*. Since *I. basta* also exhibits a higher content of bromine, we have to conclude that *I. basta* skeletons contain a higher overall number of brominated compounds than *A. cavernicola*. The question arises whether or not these brominated compounds are the same for both Verongida sponges. In order to answer this question, GC-MS analyses were performed.

### 2.2. Skeletal Amino Acid Composition

#### 2.2.1. GC-MS Analysis of the Skeletal Amino Acid Composition

The isolated skeletons of *I. basta* were treated with a saturated Ba(OH)_2_ solution to extract the skeleton-bound amino acids and allow their analysis by GC-MS. The protocol for this extraction was initially developed for the sponge *A. cavernicola* [41]. It had to be adapted and optimized for the more resistant skeletons of *I. basta*. In contrast to *A. cavernicola*, the complete extraction of *I. basta* skeletons required a significantly higher temperature (100 °C versus 37 °C) because of the stronger hydrolysis resistance of the *I. basta* spongin fibers. This emphasizes the different characters of the two different sponge skeletons.

The dissolved amino acids were derivatized using MTBSTFA and analyzed as described for *A. cavernicola* [41]. The derivatization method was optimized such that a maximum number of amino acids with their broad range of structures is completely converted. The same method as for *A. cavernicola* was then applied for *I. basta*. Since the analysis methods for both sponges were exactly the same, the results are well comparable.

**Figure 3:**
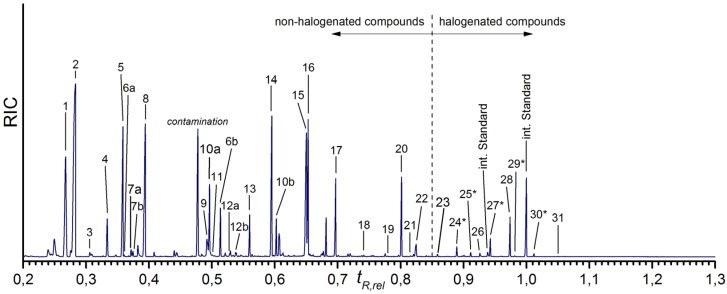
Gas chromatogram of the MTBSTFA-derivatized Ba(OH)_2_ extract of the isolated *I. basta* sponge skeletons. The relative retention time is related to the second peak of the internal standard 5-bromotryptophan. Components with * could not be verified with pure standards due to the lack of availability of these reference compounds.

The chromatogram of the GC-MS measurements is shown in Figure 3. The assignment of the peaks is given in Table 2. It was carried out as described in detail in [41], using the information in EI mass spectra and confirmed for most of the peaks by retention times of standard compounds. The structure of a few halogenated amino acids could not be verified with pure standards due to the lack of availability of these reference compounds. For these compounds, the identification was based on their mass spectra and is also described in detail in [41].

Obviously, the isolated *I. basta* sponge skeletons contain a variety of amino acids. The gas chromatogram can be divided into two parts: below a relative retention time of *t_R,rel_* = 0.85, twenty-two different amino acids could be observed. Seventeen of these amino acids could be assigned to proteinogenic amino acids. Some of the amino acids give rise to multiple peaks. This is caused by different reasons. Serine (peaks 6a, 6b) and hydroxyproline (peaks 10a, 10b) exhibit two peaks due to different degrees of derivatization. In each case, the first peak is caused by the incomplete derivatization of the molecule while the second peak is caused by the completely derivatized amino acid. Isoleucine (peaks 7a, 7b) and threonine (peaks 12a, 12b), however, yield two peaks of the same TBDMS derivative. As described previously (see [41]), the existence of two stereocenters in isoleucine as well as in threonine leads to two different diastereomers [49,50], which can be separated by gas chromatography.

The largest peak of the non-halogenated amino acids is due to glycine (peak 2). Furthermore, the proteinogenic amino acids alanine (peak 1), leucine (peak 5), proline (peak 8), aspartic acid (peak 14), glutamic acid (peak 15), lysine (peak 17), and tyrosine (peak 20) occur in larger quantities. In contrast, valine (peak 4), serine (peaks 6a, 6b), and phenylalanine (peak 13) have lower intensities. Isoleucine (peaks 7a, 7b), methionine (peak 11), threonine (peaks 12a, 12b), arginine (peak 18), histidine (peak 19), and tryptophan (peak 21) occur in the smallest amounts, where methionine, histidine and tryptophan are only found as traces.

Additionally, the non-proteinogenic amino acids AABA (peak 3), oxoproline (peak 9), hydroxyproline (peaks 10a, 10b), hydroxylysine (peak 22), as well as ornithine (peak 16), could be detected in the Ba(OH)_2_ extract of the *I. basta* skeletons. Ornithine (peak 16) occurs in a large amount, while hydroxyproline (peaks 10a, 10b) is present in smaller amounts. The other non-proteinogenic amino acids AABA (peak 3), oxoproline (peak 9) and hydroxylysine (peak 22), exhibit the smallest amounts.

Various halogenated amino acids occur at relative retention times larger than 0.85. Nine different halogenated amino acids can be identified, all of them are tyrosine derivatives. The monohalogenated amino acids 3-monochlorotyrosine (peak 23), monobromotyrosine (peak 24*) and 3-monoiodotyro-sine (peak 26), the purely dihalogenated amino acids dichlorotyrosine (peak 25*), 3,5-dibromo-tyrosine (peak 28), and 3,5-diiodotyrosine (peak 31) as well as the mixed halogenated amino acids monobromo-monochlorotyrosine (peak 27*), monochloro-monoiodotyrosine (peak 29*), and monobromo-monoiodotyrosine (peak 30*) can be detected. 3,5-dibromotyrosine (peak 28) represents the main component of the halogenated amino acids. Furthermore, monobromo-monochlorotyrosine (peak 27*) also exhibits a relatively high amount. All other halogenated tyrosines are present in smaller amounts; the lowest concentrations are found for monochloro-monoiodotyrosine (peak 29*) and 3,5-diiodotyrosine (peak 31).

#### 2.2.2. Comparison of the Found Amino Acid Composition with the Literature

The found amino acid composition of the *I. basta* skeletons was compared with the previously determined amino acid composition of *A. cavernicola* skeletons [41] from the order Verongida, and with that of the sponges *H. equina* [39] as well as *S. officinalis obliqua* [40], both belonging to the order Dictyoceratida. All these sponges are from the Demospongia class. The results are given in Table 3.

Since the extraction of the amino acids was carried out with an alkaline Ba(OH)_2_ solution in all cases, the amino acid compositions are well comparable. First of all, it can be stated that the skeletal amino acid composition of the Verongida sponges *I. basta* and *A. cavernicola* is relatively similar. There are, however, also some differences: *I. basta* skeletons exhibit the non-halogenated amino acids hydroxylysine and isoleucine, which cannot be found in *A. cavernicola* skeletons. Monobromohistidine, in turn, cannot be detected in *I. basta* skeletons but are in the skeletons of *A. cavernicola*. Striking differences occur between the amino acid composition of the two Verongida sponges and the Dictyoceratida sponges *H. equina* and *S. officinalis obliqua*, especially with respect to halogenated amino acids. Most of the characteristic halogenated amino acids are only observed in the skeletons of the Verongida sponges *I. basta* and *A. cavernicola*. The halogenated amino acids monobromotyrosine, 3-monochlorotyrosine, monochloro-monoiodotyrosine, monobromo-monochlorotyrosine and dichlorotyrosine could not be identified in *H. equina* and *S. officinalis obliqua*. Interestingly, the amino acid monobromo-monochlorotyrosine is occurring in the gastropod mollusk *Buccinum undatum* [51].

In addition, the results of the analysis of *I. basta* skeletons support the hypothesis [41] that the presence of AABA or GABA is species-specific. *H. equina* is the only sponge in which GABA occurs. In the other sponges AABA was found instead of GABA. Moreover, cystine only occurs in *H. equina* but not in the other examined species.

The proteinogenic amino acids alanine, aspartic acid, glutamic acid, glycine, leucine, lysine, proline, tryptophan, tyrosine, valine and the halogenated amino acid 3,5-diiodotyrosine have been identified in the skeletons of *I. basta* as well as in *A. cavernicola*, in the sponge *H. equina* and in the sponge *S. officinalis obliqua*. Moreover, the non-proteinogenic amino acids hydroxyproline, oxoproline and ornithine were found in all four sponge samples. The presence of hydroxyproline is well known for collagen [52]. This observation, together with the very high amount of glycine in both sponge skeletons, emphasizes the collagenous character of spongin. Oxoproline and ornithine were also found in *Aplysina aerophoba* [53], which is the closest relative to *A. cavernicola*.

Since both *H. equina* and *S. officinalis obliqua* are members of the order Dictyoceratida while *I. basta* and *A. cavernicola* belong to the order Verongida it is quite possible that the observed amino acid composition is genus specific.

For further comparison, the relative amounts of the detected amino acids were estimated for *I. basta* and *A. cavernicola* which were analyzed exactly by the same method by referring the individual peak intensities to that of the most intensive peak (cf. Table 4). Glycine was the most abundant amino acid in both sponge skeletons. Alanine, lysine, ornithine, proline, and tyrosine were present in large amounts in both samples. In contrast, phenylalanine and valine occurred in smaller amounts in both cases. Methionine, arginine and tryptophan were only detected in minute amounts.

However, the compositions of the sponge skeletons from *I. basta* and *A. cavernicola* also exhibit interesting differences with respect to the non-halogenated amino acids. Histidine, serine, and threonine were present in much larger amounts in *A. cavernicola* skeletons than in *I. basta*. For aspartic acid, glutamic acid and phenylalanine the situation is opposite. Much larger amounts of these amino acids were detected in the skeletons of *I. basta* than in *A. cavernicola*. Moreover, the amino acids hydroxylysine and small amounts of isoleucine were found in the *I. basta* skeletons, with hydroxylysine supporting the collagenous nature of *I. basta* spongin. These amino acids were not identified in the skeletons of *A. cavernicola*. Furthermore, the amount of leucine is much higher in the skeletons of *I. basta* than in *A. cavernicola*.

Within the group of halogenated amino acids, 3,5-dibromotyrosine and monobromo-monochlorotyrosine were most abundant. Smaller amounts were detected for all iodated tyrosines. The major difference was recognized in the dichlorotyrosine amounts, which were larger in the skeletons of *A. cavernicola*.

## 3. Materials and Methods

### 3.1. Sponge Samples

Sponges were collected in July 2013 while snorkeling at depths ranging from 8 to 12 m at Western Shoals, Apra Harbor, Guam (13.27.018 N; 144.39.120 E). Specimens were inspected in regards to their health and only healthy and intact specimens were collected. Sponges were kept in large Ziploc bags during collection and transported in a cooler to the Guam marine laboratories. Specimens were frozen at −20 °C and freeze-dried prior to their transport to the laboratories in Dresden, Germany (see also [20]).

### 3.2. Extraction of the Skeletons

*I. basta* skeletons were isolated following the protocol described previously [41]. Small pieces of *I. basta* (5–10 cm^2^) were soaked in 40 mL of distilled water for two weeks. Subsequently, the samples were transferred into freshly distilled water for 24 h. This procedure was repeated two times under continuous shaking.

### 3.3. Light Microscopy

Small pieces of the purified chitin-scaffolds were put on a sample holder. Microscopic studies were carried out on a Keyence BZ-8000K microscope (Keyence, Osaka, Japan). The exposure time was 1/230 s.

### 3.4. Scanning Electron Microscopy (SEM) and Energy Dispersive X-ray Spectroscopy (EDX)

Dried pieces of the skeletons were fixed on a sample holder and coated with carbon. The SEM images were recorded on a ZEISS DSM 982 GEMINI field emission scanning electron microscope (ZEISS, Oberkochen, Germany) using an acceleration voltage of 2 kV. Furthermore, EDX spectra were recorded with an acceleration voltage of 15 keV.

### 3.5. Extraction of the Chitin-Based Scaffold

The pure chitin skeletons were uncovered by an alkaline extraction according to [26]. Five to Thirty-five mg of isolated and freeze dried samples were treated with 2.5 M NaOH at 37 °C for 7 days. The remaining fibrous skeletal material was neutralized. In a second step, the samples were treated with 20% acetic acid at 37 °C for 24 h. Subsequently, the remaining fibrous skeleton material was neutralized and freeze dried again. The percentage of chitin in the skeletons was determined gravimetrically.

### 3.6. Estimation of the Content of Other Saccharides

The content of saccharides beside chitin in the skeletons was estimated using the resorcin method [47]. Therefore, 4 mg of *A. cavernicola* or 1.5 mg of *I. basta* were soaked with 1 mL pure water, 1 mL of a solution of resorcin (6 mg/mL) and 5 mL of sulfuric acid (75%) for 40 min at 95 °C and then quenched for 30 min in a darkened water bath. For calibration, soaked standard solutions of a 1:1:1 mixture of glucose, glucuronic acid and mannose containing defined amounts of chitin and protein (lysozyme) to simulate the matrix of skeletons were used. For quantification, the UV/Vis spectrometer Cary 50 (Varian, Palo Alto, CA, USA) was used with the following conditions: wavelength 300–800 nm, 0.0125 s per scan, data interval 1 nm, scan rate 4800 nm.

### 3.7. Determination of Calcium, Silicon and Sulfur Contents by ICP-OES

Digestion for calcium and sulfur determination: 2–18 mg of the skeleton were weighted into a micro vessel and digested with 450 μL of a mixture of HNO_3_ (65%), HF (47%–51%) and HCl for 15 min using microwaves at 1600 W and stepwise heating to 130 °C.

Complexation: 1.5 mL of a saturated solution of H_3_BO_3_ was added to the digested sample for complexing fluorides. Complexation was accomplished as follows: power 800 W, temperature 110 °C, time 10 min.

Digestion for silicon determination: 3 mL of HNO_3_ (65%) and 2 mL H_2_O_2_ were added to 30 mg of sponge skeletons. Microwave digestion was accomplished using the following parameters: at the beginning 400 W at 50 °C, followed by stepwise heating to 180 °C using 800 W in 60 min.

Finally, all digested samples were filled up to 10 mL with ultrapure water.

Measurement: The samples were measured with an ICP optical emission spectrometer Perkin–Elmer Optima 7000 DV using the analytical lines at 317.933 nm for calcium, at 180.669 nm for sulfur and at 212.412 nm, 251.611 nm and 288.158 nm for silicon. The following operating parameters were used: plasma argon 15 L/min, auxiliary argon 0.2 L/min, nebulizer argon 0.65 L/min, RF-power 1300 W and pump rate 1.3 L/min.

### 3.8. Ba(OH)_2_ Extraction of the Amino Acids

30 mg of the dried isolated skeletons were treated with 7.5 mL of saturated Ba(OH)_2_ solution containing 2 mg 5-bromotryptophan as internal standard at 100 °C for 5 days. Subsequently, the Ba(OH)_2_ solution was neutralized with H_2_SO_4_ and centrifuged. The supernatant was removed and freeze dried.

### 3.9. GC-MS Measurements of the Skeleton Extracts

One mg from each of the dried Ba(OH)_2_ extracts was soaked in 20 μL 2.5 M HCl and dried under a gentle stream of nitrogen. Subsequently, the residues were soaked twice in 40 μL EtOH and dried under nitrogen. Fifty μL acetonitrile and 50 μL MTBSTFA were added to the dry residues. The mixtures were sonicated for 30 s and then heated to 70 °C for 30 min. One μL of the resulting solution was injected into the GC-MS.

Analyses were carried out on an Agilent Technologies 6890N gas chromatograph directly coupled to an Agilent Technologies 5973N mass spectrometer (Agilent Technologies, Santa Clara, CA, USA). GC separations were performed on a SPB^®^-5 capillary GC column (Sigma-Aldrich, St. Louis, MO, USA). The flow of helium as carrier gas was 1 mL/min. The injector temperature was 300 °C. Split/Splitless injection was used (splitless time 1 min). The column temperature was programmed as followed: isothermal 115 °C for 3 min, then heating up to 300 °C at a rate of 4 K/min, then isothermal 300 °C for 30 min. The ion source temperature was 250 °C and the transfer line temperature was 300 °C. The mass spectra were recorded in the electron impact (EI) ionization mode at 70 eV, *m*/*z* range 70–850. The chromatograms were recorded after 3 min solvent delay. The retention time *t*_R_ was normalized to the second peak of the internal standard. The intensity was normalized to the weighted sample and the total intensity of both peaks of the internal standard.

## 4. Conclusions

The isolated skeleton of the marine demosponge *I. basta* was analyzed with respect to its amino acid composition. These investigations revealed that the skeleton of *I. basta* contains 17 proteinogenic amino acids as well as five different non-proteinogenic amino acids. Moreover, we detected nine halogenated amino acids, all of them are halogenated tyrosines.

In conclusion, we can state the following:

1. The composition of non-halogenated amino acids in *I. basta* is similar to that of *A. cavernicola*. Abundant amino acids such as glycine and hydroxyproline confirm the collagenous nature of the *I. basta* spongin in analogy to all other investigated sponges.
2. *I. basta* exhibits a similar variety of halogenated amino acids as already observed for *A. cavernicola*—in contrast to the Dictyoceratida sponges *H. equina* and *S. officinalis obliqua.* It is, therefore, tempting to speculate that this variety of halogenated amino acids is characteristic for the order Verongida.
3. The differences in amino acid composition in the sponge skeletons of *I. basta* and *A. cavernicola* clearly show that the spongin in the skeletons of Verongid sponges is similar, but also exhibits some characteristic differences.
4. Further investigations of the amino acid composition of other sponge samples should be performed in the future to include a larger set of different sponge species into this comparison.

## Figures and Tables

**Table 1:** General chemical characterization of the skeletons of *I. basta* in comparison to the skeletons of *A. cavernicola*.

Parameter	*I. basta*	*A. cavernicola*
percentage of skeleton in the sponge	50.1 ± 20.0 wt. %	3.1 ± 1.3 wt. %
percentage of chitin in the skeleton	17.1 ± 1.4 wt. %	8.0 ± 1.4 wt. %
percentage of other saccharides in the skeleton	3–4 wt. %	1–2 wt. %
content of calcium in the skeleton	15.5 mg/g	3.5 mg/g
estimated content of calcium carbonate in the skeleton	3.9 wt. %	0.9 wt. %
content of silicon in the skeleton	<1.7 mg/g	<1.7 mg/g
estimated content of protein in the skeleton	≤77 wt. %	≤90 wt. %
content of sulfur in the skeleton	14.4 mg/g	11.8 mg/g
halogens present in the skeleton	Br, Cl, I	Br, Cl, I
bromine content in the skeleton [44]	51 ± 4 mg/g	40 ± 3 mg/g

**Table 2:** Amino acids detected as TBDMS-derivatives in the Ba(OH)_2_ extract of isolated *I. basta* sponge skeletons. Components with * could not be verified with pure standards due to the lack of availability of these reference compounds.

Peak	Amino Acid	Proteinogenic	Halogenated
1	Alanine	X	
2	Glycine	X	
3	α-Aminobutyric Acid (AABA)		
4	Valine	X	
5	Leucine	X	
6a	Serine (2 TBDMS)	X	
7a	Isoleucine	X	
7b	Isoleucine	X	
8	Proline	X	
9	Oxoproline		
10a	Hydroxyproline (2 TBDMS)		
11	Methionine	X	
6b	Serine (3 TBDMS)	X	
12a	Threonine (3 TBDMS)	X	
12b	Threonine (3 TBDMS)	X	
13	Phenylalanine	X	
14	Aspartic Acid	X	
10b	Hydroxyproline (3 TBDMS)		
15	Glutamic Acid	X	
16	Ornithine		
17	Lysine	X	
18	Arginine	X	
19	Histidine	X	
20	Tyrosine	X	
21	Tryptophan	X	
22	Hydroxylysine		
23	3-Monochlorotyrosine		X
24 *	Monobromotyrosine		X
25 *	Dichlorotyrosine		X
26	3-Monoiodotyrosine		X
27 *	Monobromo-monochlorotyrosine		X
28	3,5-Dibromotyrosine		X
29 *	Monochloro-monoiodotyrosine		X
30 *	Monobromo-monoiodotyrosine		X
31	3,5-Diiodotyrosine		X

**Table 3:** Comparison of detected amino acids with the known amino acid composition of spongin reported in the literature. Amino acids which are only found in *I. basta* are marked in orange; those only found in *A. cavernicola* are marked in green.

Halogenation State	Amino Acid	*Ianthella basta*	*Aplysina cavernicola* [41]	*Hippospongia equina* [39]	*Spongia officinalis obliqua* [40]
	Alanine	X	X	X	X
Non-halogenated	α-Aminobutyric Acid (AABA)	X	X		X
	γ-Aminobutyric Acid (GABA)			X	
	Arginine	X	X	X	
	Aspartic Acid	X	X	X	X
	Cystine			X	
	Glutamic Acid	X	X	X	X
	Glycine	X	X	X	X
	Histidine	X	X	X	
	Hydroxylysine	X			
	Hydroxyproline	X	X	X	X
	Isoleucine	X			
	Leucine	X	X	X	X
	Lysine	X	X	X	X
	Methionine	X	X		X
	Ornithine	X	X	X	
	Oxoproline	X	X		
	Phenylalanine	X	X	X	
	Proline	X	X	X	X
	Serine	X	X	X	
	Threonine	X	X	X	
	Tryptophan	X	X	X	X
	Tyrosine	X	X	X	X
	Valine	X	X	X	X
Halogenated	Monobromohistidine		X		
	Monobromotyrosine	X	X		
	3-Monochlorotyrosine	X	X		
	3-Monoiodotyrosine	X	X		X
	Monochloro-monoiodotyrosine	X	X		
	Monobromo-monochlorotyrosine	X	X		
	Monobromo-monoiodotyrosine	X	X		
	Dichlorotyrosine	X	X		
	3,5-Dibromotyrosine	X	X		X
	3,5-Diiodotyrosine	X	X	X	X

**Table 4:** Comparison of the relative amino acid amounts in *I. basta* and *A. cavernicola* skeletons. Relative amounts were estimated referring the peak heights in the gas chromatogram to the most intense peak (++++: very large amount (100%–80%); +++: large amount (80%–30%); ++: small amount (30%–10%); +: smallest amount (<10%); -: not present). Amino acids only found in *I. basta* are marked in orange, those only found in *A. cavernicola* are marked in green.

Halogenation State	Amino Acids	Amounts in	
		*Ianthella basta*	*Aplysina cavernicola* [41]
Non-halogenated	Glycine	++++	++++
	Alanine	+++	+++
	Aspartic Acid	+++	++
	Glutamic Acid	+++	++
	Hydroxyproline	+++	+++
	Leucine	+++	+
	Lysine	+++	+++
	Ornithine	+++	+++
	Proline	+++	+++
	Tyrosine	+++	+++
	Oxoproline	++	+
	Phenylalanine	++	+
	Serine	++	++++
	Valine	++	++
	Arginine	+	+
	α-Aminobutyric Acid (AABA)	+	+
	Histidine	+	++
	Hydroxylysine	+	-
	Isoleucine	+	-
	Methionine	+	+
	Threonine	+	+++
	Tryptophan	+	+
	Monobromo-monochlorotyrosine	++	+++
	3,5-Dibromotyrosine	++	+++
	Monobromotyrosine	+	++
Halogenated	3-Monochlorotyrosine	+	+
	3-Monoiodotyrosine	+	+
	Monochloro-monoiodotyrosine	+	+
	Monobromo-monoiodotyrosine	+	+
	Dichlorotyrosine	+	++
	3,5-Diiodotyrosine	+	+
	Monobromohistidine	-	+
